# Supplementary material for: Occurrence of major infectious diseases and healthcare seeking among young children with disabilities in Sierra Leone using cross-sectional population-based survey data
Source: BMJ Paediatr Open. 2024 Jun 25;8(1):e002460. doi: 10.1136/bmjpo-2023-002460 (PMC11202746; doi:10.1136/bmjpo-2023-002460)
Supplement: Author's manuscript [file bmjpo-2023-002460.draft_revisions.pdf]

BMJ Paediatrics Open

BMJ Paediatrics Open is committed to open peer review. As part of this commitment we make the peer review history of every article we publish publicly available.

When an article is published we post the peer reviewers' comments and the authors' responses online. We also post the versions of the paper that were used during peer review. These are the versions that the peer review comments apply to.

The versions of the paper that follow are the versions that were submitted during the peer review process. They are not the versions of record or the final published versions. They should not be cited or distributed as the published version of this manuscript.

BMJ Paediatrics Open is an open access journal and the full, final, typeset and author-corrected version of record of the manuscript is available on our site with no access controls, subscription charges or pay-per-view fees (http://bmjpaedsopen.bmj.com).

If you have any questions on BMJ Paediatrics Open's open peer review process please email info.bmjpo@bmj.com

BMJ Paediatrics Open

The occurrence of major infectious diseases and health care seeking among young children with disabilities in Sierra Leone using cross-sectional population-based survey data

Journal:	*BMJ Paediatrics Open*
Manuscript ID	bmjpo-2023-002460
Article Type:	Original research
Date Submitted by the Author:	15-Dec-2023
Complete List of Authors:	Ekman, Anna-Theresia; Karolinska Institutet, Global Public Health<br>Cherry, Esagila; Linkoping University Department of Health Medicine and Caring Sciences<br>Sengeh, Paul; FOCUS1000<br>Webber, Nance; FOCUS1000<br>Jalloh, Mohammad; FOCUS1000<br>Orsini, Nicola; Karolinska Institutet, Global Public Health<br>Alfvén, Tobias; Karolinska Institutet, Global Public Health<br>Frielingsdorf Lundqvist, Helena; Linkoping University Department of Health Medicine and Caring Sciences
Keywords:	Epidemiology, Mortality, Neurology

SCHOLARONE™
Manuscripts

I, the Submitting Author has the right to grant and does grant on behalf of all authors of the Work (as defined in the below author licence), an exclusive licence and/or a non-exclusive licence for contributions from authors who are: i) UK Crown employees; ii) where BMJ has agreed a CC-BY licence shall apply, and/or iii) in accordance with the terms applicable for US Federal Government officers or employees acting as part of their official duties; on a worldwide, perpetual, irrevocable, royalty-free basis to BMJ Publishing Group Ltd ("BMJ") its licensees and where the relevant Journal is co-owned by BMJ to the co-owners of the Journal, to publish the Work in this journal and any other BMJ products and to exploit all rights, as set out in our licence.

The Submitting Author accepts and understands that any supply made under these terms is made by BMJ to the Submitting Author unless you are acting as an employee on behalf of your employer or a postgraduate student of an affiliated institution which is paying any applicable article publishing charge ("APC") for Open Access articles. Where the Submitting Author wishes to make the Work available on an Open Access basis (and intends to pay the relevant APC), the terms of reuse of such Open Access shall be governed by a Creative Commons licence – details of these licences and which Creative Commons licence will apply to this Work are set out in our licence referred to above.

Other than as permitted in any relevant BMJ Author's Self Archiving Policies, I confirm this Work has not been accepted for publication elsewhere, is not being considered for publication elsewhere and does not duplicate material already published. I confirm all authors consent to publication of this Work and authorise the granting of this licence.

Confidential: For Review Only

The occurrence of major infectious diseases and health care seeking among young children with disabilities in Sierra Leone using cross-sectional population-based survey data

Abstract

Background: Children with disabilities are at risk of worse health outcomes compared to children without functional difficulties. Sierra Leone has one of the worlds highest prevalences of functional difficulties among children, but little is know about the co-occurrence of major infectious diseases and health care seeking behaviours among children with disabilities.

Methods: We used household survey cross-sectional data on children two to four years old and logistic regression models estimating odds ratios (ORs) between functional difficulties and symptoms of infectious diseases including diaorrhea, fever and acute respiratory infection, adjusted for sex, age, and stunting. We also examined whether caregivers sought advice or treatment for the illness from any source and if the child was given any treatment for the illness.

Results: There was an increased risk of fever among children with functional difficulty (AOR = 1.3, 95% CI 1.1 - 1.8) and children with severe functional difficulty (AOR = 1.6, 95% CI 1.0-2.7). Children with severe functional difficulty were also at increased risk of diarrhea (AOR = 1.8, 95% CI = 1.1 – 3.3). There were no significant differences in seeking advice or treatment for diarrhea, fever or ARI symptoms between the groups.

Conclusions: In Sierra Leone, children with functional difficulties, especially severe functional difficulties, more often have symptoms of major childhood diseases that are known to increase under-five mortality.

Key message

What is already known on this topic: Functional difficulty among young children is common in Sierra Leone. Even so, there is lacking information on major infectious diseases and health care seeking among children with functional difficulties in Sierra Leone.

What this study adds: We found that the odds of fever was 30% higher among children with functional difficulty (AOR = 1.3, 95% CI 1.1 - 1.8), and 60% higher among children with

Confidential: For Review Only

severe functional difficulty (AOR = 1.6, 95% CI 1.0-2.7). For children with severe functional difficulties, it was also more common with diarrhea (AOR = 1.8, 95% CI = 1.1 – 3.3).

How this study might affect research, practice or policy: To overcome these health inequities, efforts must be made to include children with disabilities in preventive and health promoting child health as well as curative care, while acknowledging that targeted interventions tailored to the needs of children with disabilities will be of additional benefit.

Confidential: For Review Only

Introduction

Almost 240 million children worldwide have functional difficulties, with the highest prevalence in West Africa (1). Persons with disability experience significantly poorer health than their non-disabled peers, ultimately risking a shortened lifespan (2-5). To meet the 2030 Agenda pledge of leaving no one behind (6), an increased understanding of health and health-seeking behavior among children with disabilities is needed.

Five million children in the world still die before their fifth birthday, with pneumonia, diarrhea, and malaria being the leading causes of under-five mortality after the neontal period (7). All conditions are largely preventable and treatable through cost-effective measures, and malnutrition is an important risk factor (7).

In Sierra Leone, the under-five mortality rate is 109 deaths per 1000 live births (8). Malnutrition is widespread, with 30% of children below five being stunted, 5% being wasted, and 14% being underweight in 2019 (9). The lack of health information on children living with disabilities in Sierra Leone has previously been noted (10).

The child functioning module (CFM) was introduced during the 2017 Sierra Leone Multiple Indicator Cluster Survey (MICS), as a novel tool to collect data on functional difficulties among children. In line with the biopsychosocial model, the CFM collects data on functional difficulties rather than specific biomedical impairments. The underlying impairments captured by the tool are heterogeneous and include both mild to severe impairments. The 2017 Sierra Leone MICS also provides information on leading causes of under-five mortality, measured through episodes of fever (proxy for malaria), acute respiratory infection (ARI) symptoms (proxy for pneumonia) and diarrhea (11).

Enhancing knowledge on the relationship between disability and leading causes of under five mortality can inform health planning policy to accurately reflect the needs of children, ultimately improving life length and quality for a vulnerable population. The aim of this article is therefore to examine the association between three symptoms of infectious diseases and disability among young children in Sierra Leone, compared to children without disabilities. A secondary aim is to study health care seeking behavior and access to treatment for the same symptoms in the same population.

Confidential: For Review Only

Methods

Study design and description of the data set

This study utilizes two staged cluster-survey data from the 2017 Sierra Leone MICS. The survey included 11,774 children under five of whom 11,764 mothers/caretakers were interviewed (response rate 99.9%) (16). Data on functional difficulties was collected from children two to four years and formed our study population, in total 7117 children. Sample allocation and participant selection have been described in detail (12). Data were extracted from the dataset for children under five, including information about functional difficulties from the CFM (Figure 1), and information about disease episodes, care-seeking, and access to treatment from the Care of Illness module (Figure 2).

Operationalizing disability

The CFM estimates children at risk of disability through a Likert-like scale, collecting information on functional difficulties in the functional areas of seeing, hearing, mobility, dexterity, communication, learning, behavior, and playing, using primary caregiver (usually mothers) as proxy-responders (13). Children with functional difficulties were defined as those with a lot of difficulties and/or who cannot function at all in one or more of the different domains of functioning (Figure 1). A child with severe functional difficulty was defined as 'cannot do at all' in any of the domains. The only exception was the domain of controlling behavior, where a child was considered having a functional difficulty at the response of 'A lot more'.

Confidential: For Review Only

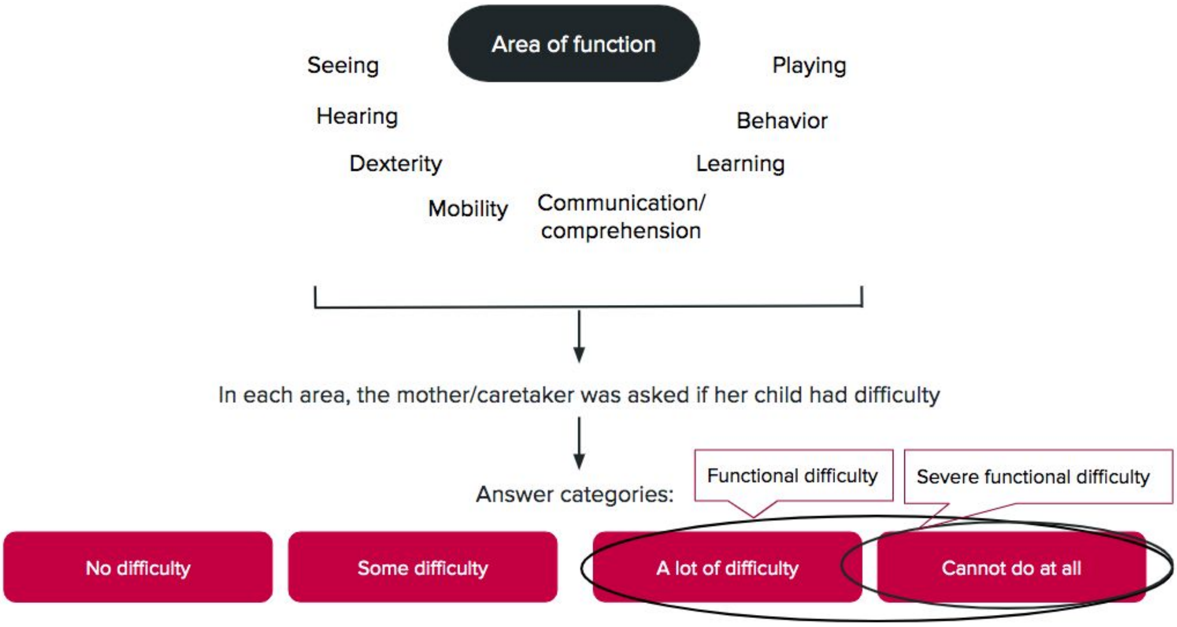

Figure 1 - Identifying children with functional difficulties
The child functioning module asked the mother/caretaker a set of questions on eight areas of functioning regarding their child. Each area was assessed against a rating scale, with the answer categories 'No difficulty', 'Some difficulty', 'A lot of difficulty' and 'Cannot do at all'. The child was considered having a functional difficulty at the response of 'A lot of difficulty' or 'Cannot do at all', and severe functional difficulty at the response of 'Cannot do at all'. The only exception was the domain of controlling behavior, where the response categories were instead 'Not at all', 'Less', 'The same', 'More' and 'A lot more' (not presented in the figure). The child was considered having a functional difficulty at the response of 'A lot more'.

Episodes of diarrhea, fever or respiratory difficulties

The caregiver was asked to estimate whether the child had an episode of diarrhea, fever, or ARI symptoms during the preceding two-week period. These survey questions have previously been utilized in analyses of MICS surveys (14, 15). ARI symptoms were defined as rapid or difficult breathing during illness with cough, when symptoms were perceived to be due to a problem in the chest or both a problem in the chest and a blocked or runny nose (12, 16). Thus, only having symptoms of an upper respiratory tract infection were not considered as ARI symptoms. If the child had either diarrhea, fever or ARI symptoms, the caregiver was asked if they had sought any advice or treatment for the illness from any source and if the child was given any medicine to treat the illness (Figure 2).

For Peer Review Only

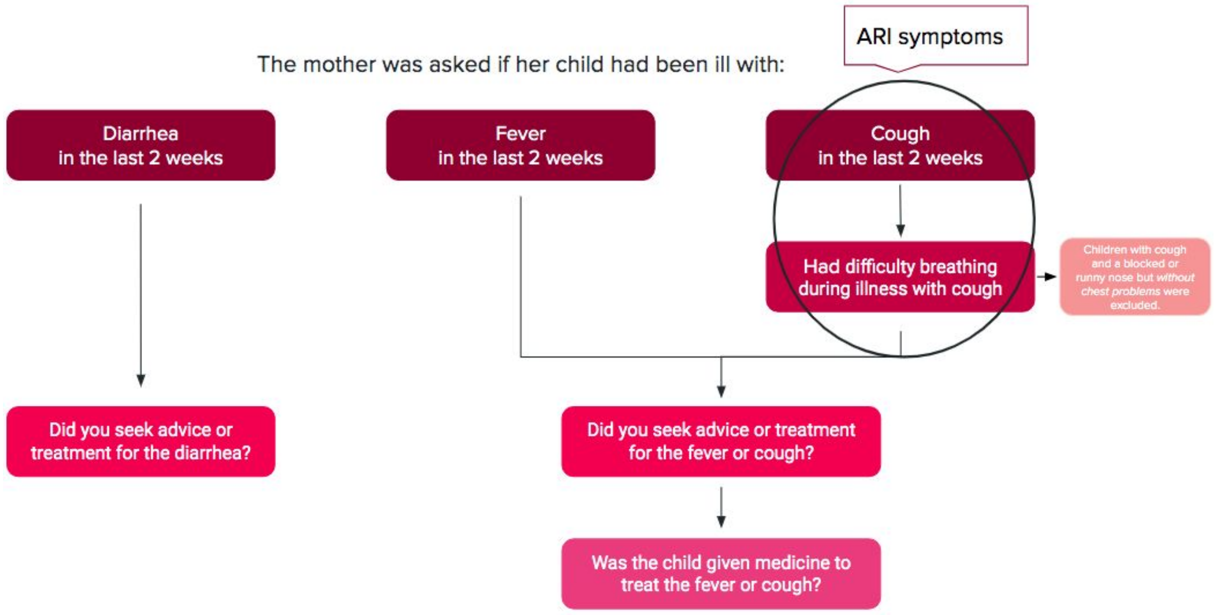

Figure 1 - Identifying episodes of illness, healthcare seeking and access to treatment
In the survey's Care of Illness (CA) module, the mother/caretaker was asked if the child had been ill with diarrhea, fever or cough in the last 2 weeks. If the child had been ill with cough, the mother/caretaker was asked if the child had difficulty breathing during that illness. The child was considered having ARI symptoms if it had cough and difficulty breathing at the same time, meaning cough due to a chest problem. To identify healthcare seeking behavior, the mother/caretaker was asked if they had sought advice or treatment for the illnesses respectively. To identify access to treatment, the mother/caretaker was asked if the child had been given medicine for the fever or cough.

Statistical Analysis

Functional difficulty/severe functional difficulty were used as independent variables. Potential predictors considered were child age, child gender, stunting, household wealth, maternal level of education, and rural-urban residence status. Crude (OR) and adjusted odds ratios (AOR) with 95% confidence intervals (CI) were estimated with multivariable logistic regression models. Stata version 16.0 was used for all survey analyses with the stratum variable from the household dataset and centering single units.

Ethical Approval and Consent to Participate

Ethical approval for data collection was attained from the Ethics and Scientific Review Committee in Sierra Leone by the Sierra Leone MICS team (12). Informed consent was obtained from all participants during data collection, and data has been de-identified for confidentiality. This study is a secondary analysis of anonymized data from MICS, publicy accessible for research purposes through the MICS website.

Public involvement

For Review Only

Due to this being a secondary data analysis, it was not possible to involve the public in the design, or conduct, or reporting, or dissemination plans of our research.

Results

The weighted sample included 7,090 children age 2-4 years, of which 7% were identified as having functional difficulties and almost 2% were identified as having severe functional difficulties (Table 1). One in five children (20%) had fever in the last 2 weeks, 7% had diarrhea and 2% had ARI symptoms. Functional difficulty was associated with child age, child gender and stunting, and these factors were included in the adjusted logistic regression. The odds of fever was 30% higher among children with functional difficulty (AOR = 1.3, 95% CI 1.1 - 1.8). The odds of fever was 60% higher among children with severe functional difficulty (AOR = 1.6, 95% CI 1.0-2.7). For children with severe functional difficulties, it was also significantly more common with diarrhea (AOR = 1.8, 95% CI = 1.1 – 3.3). There was no significant differance for ARI symptoms after adjusting for confounding.

For more than two thirds of the children, caregivers had sought advice or treatment, and almost all were given some form of medicine for fever or cough. There were no significant differences in seeking advice or treatment for diarrhea, fever or ARI symptoms (Table 3). However, there was a trend towards less health care seeking for diarrhea in children with functional difficulties and severe difficulties, and a trend towards more health care seeking for fever or ARI sympoms (Table 3). In this material, there were no differences between children with or without functional difficulties in odds of being given medicine to treat the illness.

Confidential: For Review Only

Table 1 – Weighted sample characteristics of the study population 2- to 4-year-olds.

**Table 1. Demography of study population (weighted)**	
Variable	Total n (%)
Total number of children	7090 (100%)
Children with functional difficulties	471 (7%)
Children with severe functional difficulties	126 (2%)
**Child factors**	
**Sex**	
Male	3504 (59)
Female	3586 (51)
**Age**	
2 years	2388 (34)
3 years	2351 (33)
4 years	2351(33)
**Malnutrition**	
Underweight	760 (11)
Stunted	2121 (30)
Wasted	221 (3)
**Caregiver factors**	
**Education level**	
Pre-primary or none	4528 (64)
Primary	853 (12)
Junior Secondary	875 (12)
Senior Secondary or Higher	834 (12)
**Household and enviromental factors**	
**Area**	
Urban	2663 (38)
Rural	4426 (62)
**Wealth**	
Poorest	1679 (24)
Second	1595 (23)
Middle	1482 (21)
Fourth	1222 (17)
Richest	1112 (16)
**Common childhood illnesses**	
Occurence of illness during the preceeding two weeks	
Diarrhoea	480 (7)
Fever	1460 (21)
**Cough**	1157 (16)
**Difficulty breathing during illness with cough**	452 (6)

Confidential: For Review Only

**Acute respiratory infection**	**111 (2)**
Health-care seeeking behavior	
Sought advice or treatment for diarrhoea from any source	336 (70)
**Sought advice or treatment for the fever or acute respiratory infection**	**1070 (72)**
Access to treatment	
Given any type of treatment for diarrhoea	414 (86)
**Given medicine to treat the fever or acute respiratory infection**	**1348 (91)**

Confidential: For Review Only

Table 2 - Weighted odds ratios of infectious diseases in children with and without functional difficulty.

Table 2. Weighted odds ratios of infectious diseases in chidlren with and without functional difficulty				
	Functional difficulties		Severe functional difficulties	
	Odds ratio (95% CI*)	Adjusted** odds ratio (95% CI)	Odds ratio (95% CI)	Adjusted** odds ratio (95% CI)
Occurence of illness during the preceeding two weeks				
Diarrhoea	1.4 (1-2)	1.3 (.9 - 1.8)	2 (1.1- 3.6)	1.8 (1 - 3.3)
Fever	1.4 (1-1.9)	1.3 (1 - 1.8)	1.7 (1 - 2.7)	1.6 (1 - 2.7)
Cough	1 (.8-1.4)	.9 (.7 - 1.3)	2 (1.2 - 3.5)	2 (1.1 - 3.3)
Difficulty breathing during illness with cough	1.4 (1-2)	1.3 (.9 - 2)	1.7 (.8 - 3.8)	1.7 (.7 - 3.7)
Acute respiratory infection	1.8 (1 - 3.5)	1.7 (.9 - 3.3)	2.6 (.9 - 7.2)	2.4 (.8 - 7)

*CI = Confidence Intervals. ** Adjusted for child's age, sex, and stunting.

Table 3 – Weighted odds ratios of healthcare seeking behavior in children with and without functional difficulty.

Table 3. Weighted odds ratios of health-care seeking behavior in children with and without functional difficulty				
	Functional difficulties		Severe functional difficulties	
	Odds ratio (95% CI*)	Adjusted** odds ratio (95% CI)	Odds ratio (95% CI)	Adjusted** odds ratio (95% CI)
Health-care seeking behavior				
Sought advice or treatment for diarrhoea from any source	.8 (.4 -1.6)	.8 (.4 - 1.6)	.9 (.3-3)	.9 (.3 - 3.1)
Sought advice or treatment for the fever or acute respiratory infection	1.2 (.7 - 2.1)	1.2 (.7 - 2)	1.3 (.5 - 3.7)	1.3 (.5 - 3.8)
Access to treatment				
Given any type of treatment for diarrhoea	.8(.3-1.9)	1 (.4 - 2.5)	1 (.2 - 5)	1.3 (.2 - 6.6)
Given medicine to treat the fever or acute respiratory infection	1 (.5 - 2.2)	1.1 (.6 - 2.4)	1.1 (.3 - 4.3)	1.3 (.3 - 4.7)

*CI = Confidence Intervals. ** Adjusted for child's age, sex, and stunting.

Discussion

In this study, children with functional difficulties were more prone to experience episodes of fever, and children with severe functional difficulties were significantly more likely to be ill with diarrhea, compared to children without functional difficulty. We found no significant difference on seeking or accessing treatment between children with or without functional difficulties.

Several conditions leading to functional difficulties, such as autism spectrum disorder, ADHD, cerebral palsy, and seizures/epilepsy, are associated with somatic, psychiatric, and neurological comorbidities (17). For example, children with cerebral palsy often have dysphagia, which places them at risk for aspiration pneumonia (18). This may be part of the reason behind the association between functional difficulty and the examined symptoms and infectious diseases.

Even though it is not possible to understand the underlying impairments it is likely that children with severe functional difficulties have more outspoken conditions, such as severe cerebral palsy. Indeed, a recent article investigating the same study population found a high risk of co-morbidity between difficulties in different functional domains (19), which reflects that some children within the group are more affected than others.

Another possible mechanism of association may be malnutrition. The relationship between malnutrition and childhood disability has been confirmed in multiple studies (20), and malnutrition is a shared risk factor for pneumonia, diarrhea and malaria (21). It has also been established that children with functional difficulties in Sierra Leone have an increased level of stunting of 38% (19). While this could explain part of the seen relationship between disability and infectious diseases, the increased odds of symptoms of infectious diseases persist after adjusting for stunting in this study population. The plethora of biomedical conditions captured by the CFM makes it difficult to fully entangle the relationship between disability, infectious diseases and malnutrition.

The increased susceptibility to major infectious diseases among children with disability has previously been described. A multilateral analysis of three to four-year-old's at risk for intellectual disability utilizing MICS data from 2010 to 2015 and data from 24 low- and middle-income countries, found an increased risk of symptoms of ARI, diarrhea, and fever

Confidential: For Review Only

(15). Although it did not comment on country-specific results, estimates for Sierra Leone were reported, showing that children at-risk of intellectual disability were significantly less likely to have diarrhea (15). Our results align with the study's pooled findings for low-income countries, but differ from the study's findings on Sierra Leone.

A secondary finding is that one in five children in Sierra Leone had fever during the previous two weeks, likely reflecting that fever is an unprecise proxy for malaria. The high proportion in this survey is in line with results from similar surveys (14).

Strengths and Limitations

This study builds on a large and nationally representative study population, and the introduction of the CFM may allow for comparison between settings and to repeat measures in similar study populations over time. However, cross-sectional data cannot be used to draw conclusions about causality, and it is not possible to know what types of impairments underpin the individual child's functional difficulties.

The proxies used for the infectious illnesses may have low specificity; it has been described that not all reported ARI symptoms are in fact true pneumonia (16) and fever can be caused by other diseases than malaria. In some surveys a rapid diagnostic test for malaria is included, but this was not the case in the Sierra Leone 2017 MICS. However, all disease proxies used are well-established in household surveys. Also, the fact that caregivers report symptoms from the two weeks preceding the survey, makes it likely that estimated prevalences are susceptible to seasonal changes in disease patterns. This study focuses on the difference between groups of children which we believe are less affected by seasonal variation.

We could not document significant differences on healthcare seeking behavior or access to treatment. While this may be true, it can also be due to the relatively small number of children with functional difficulties in the dataset who had symptoms of each infectious disease. This was also the reason why the multilateral analysis mentioned above could not generate country-specific estimates of access to treatments (15).

Conclusion

In Sierra Leone, children with functional difficulties, especially severe functional difficulties, are more affected by major childhood diseases that are known to increase under five

Confidential: For Review Only

mortality. To overcome these health inequities, effort must be made to include children with disabilities in preventive and health promoting child health as well as curative care, while acknowledging that targeted interventions tailored to the needs of children with disabilities will be of additional benefit. Investing in health equity could result in lowered child morbidity and mortality, ultimately fulfilling the 2030 Agenda's pledge of leaving no one behind.

Funding statement: This work was supported by Swedish regional ALF funding 2019. ALF is the abbreviation in Swedish of an agreement between the central government and seven regions on physician education and clinical research. The data collection was done through the Multiple Indicator Cluster Survey and not funded through the study.

Competing interests statement: HF has received ALF funding for other studies 2022. Otherwise none to declare.

Authors contribution: AE and EC contributed equally to the work. AE, EC and HF conceptualized the idea and designed the study. PS, NW, MBH, NO, and TA provided input on the proposed research questions as well as methodology. All authors contributed to the subsequent drafts. All authors approved the final manuscript as submitted and agreed to be accountable for all aspects of the work.

Data sharing statement: Data are available through the UNICEF multiple indicator cluster survey webpage (https://mics.unicef.org/).

References

1. United Nations Children's Fund (UNICEF). Seen, Counted, Included: Using data to shed light on the well-being of children with disabilities. New York: UNICEF; 2021.
2. Namaganda LH, Almeida R, Kajungu D, Wabwire-Mangen F, Peterson S, Andrews C, et al. Excessive premature mortality among children with cerebral palsy in rural Uganda: A longitudinal, population-based study. PLoS One. 2020;15(12):e0243948.
3. Abuga JA, Kariuki SM, Kinyanjui SM, Boele Van Hensbroek M, Newton CR. Premature mortality in children aged 6-9 years with neurological impairments in rural Kenya: a cohort study. Lancet Glob Health. 2019;7(12):e1728-e35.
4. Emerson E, Savage A, Llewellyn G. Prevalence of underweight, wasting and stunting among young children with a significant cognitive delay in 47 low-income and middle-income countries. J Intellect Disabil Res. 2020;64(2):93-102.
5. Emerson E, Hatton C, Baines S, Robertson J. The physical health of British adults with intellectual disability: cross sectional study. Int J Equity Health. 2016;15:11.
6. UN General Assembly. Transforming our world: the 2030 Agenda for Sustainable Development [Internet]. New York: United Nations; 2015.
7. United Nations Inter-agency Group for Child Mortality Estimation (UN IGME). Levels & Trends in Child Mortality: Report 2022, Estimates developed by the United

Confidential: For Review Only

Nations Inter-agency Group for Child Mortality Estimation. New York: United Nations Children's Fund; 2023.
8. World Bank. Mortality rate, under-5 (per 1,000 live births) [Internet]: The World Bank Group; [updated 2019; cited 2021 Dec 12]. Available from: https://data.worldbank.org/indicator/SH.DYN.MORT.
9. Statistics Sierra Leone (Stats SL) and ICF. Sierra Leone Demographic and Health Survey 2019 [Internet]. Freetown, Sierra Leone, and Rockville, Maryland, USA: Stats SL and ICF; 2020.
10. Kabia F, Tarawally U. Sierra Leone 2015 Population and Housing Census - Thematic Report on Disability. Freetown: Statistics Sierra Leone; 2017.
11. World Health Organization. WHO Guidelines for malaria [Internet]. Geneva: World Health Organization; 2021.
12. Statistics Sierra Leone. Sierra Leone Multiple Indicator Cluster Survey 2017, Survey Findings Report. Freetown, Sierra Leone: Statistics Sierra Leone; 2018.
13. Loeb M, Mont D, Cappa C, De Palma E, Madans J, Crialesi R. The development and testing of a module on child functioning for identifying children with disabilities on surveys. I: Background. Disabil Health J. 2018;11(4):495-501.
14. Simen-Kapeu A, Bogler L, Weber A-C, Ntambi J, Zagre NM, Vollmer S, et al. Prevalence of diarrhoea, acute respiratory infections, and malaria over time (1995-2017): A regional analysis of 23 countries in West and Central Africa.pdf>. Journal of Global Health. 2021;11.
15. Emerson E, Savage A. Acute respiratory infection, diarrhoea and fever in young children at-risk of intellectual disability in 24 low- and middle-income countries. Public Health. 2017;142:85-93.
16. Campbell H, El Arifeen S, Hazir T, O'Kelly J, Bryce J, Rudan I, et al. Measuring coverage in MNCH: challenges in monitoring the proportion of young children with pneumonia who receive antibiotic treatment. PLoS Med. 2013;10(5):e1001421.
17. Akmatov MK, Ermakova T, Bätzing J. Psychiatric and Nonpsychiatric Comorbidities Among Children With ADHD: An Exploratory Analysis of Nationwide Claims Data in Germany. J Atten Disord. 2021;25(6):874-84.
18. Arvedson JC. Feeding children with cerebral palsy and swallowing difficulties. Eur J Clin Nutr. 2013;67 Suppl 2:S9-12.
19. Ekman AT, Sengeh PA, Webber N, Jalloh MB, Hollander AC, Newby H, et al. Prevalence of children under five with disabilities in Sierra Leone in 2017: Insights from a population-based multiple indicator cluster survey. Disabil Health J. 2023:101481.
20. Hume-Nixon M, Kuper H. The association between malnutrition and childhood disability in low- and middle- income countries: systematic review and meta-analysis of observational studies. Trop Med Int Health. 2018;23(11):1158-75.
21. United Nations Inter-agency Group for Child Mortality Estimation (UN IGME). Levels & Trends in Child Mortality: Report 2020 [Internet]. New York: United Nations Children's Fund; 2020.

Confidential: For Review Only

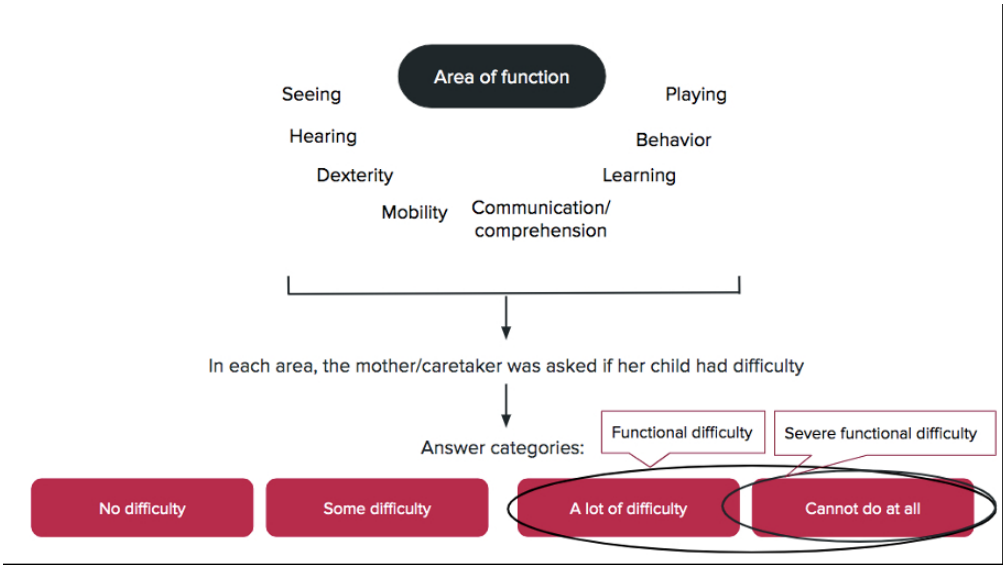

Figure 1 - Identifying children with functional difficulties

The child functioning module asked the mother/caretaker a set of questions on eight areas of functioning regarding their child. Each area was assessed against a rating scale, with the answer categories 'No difficulty', 'Some difficulty', 'A lot of difficulty' and 'Cannot do at all'. The child was considered having a functional difficulty at the response of 'A lot of difficulty' or 'Cannot do at all', and severe functional difficulty at the response of 'Cannot do at all'. The only exception was the domain of controlling behavior, where the response categories were instead 'Not at all', 'Less', 'The same', 'More' and 'A lot more' (not presented in the figure). The child was considered having a functional difficulty at the response of 'A lot more'.

160x89mm (144 x 144 DPI)

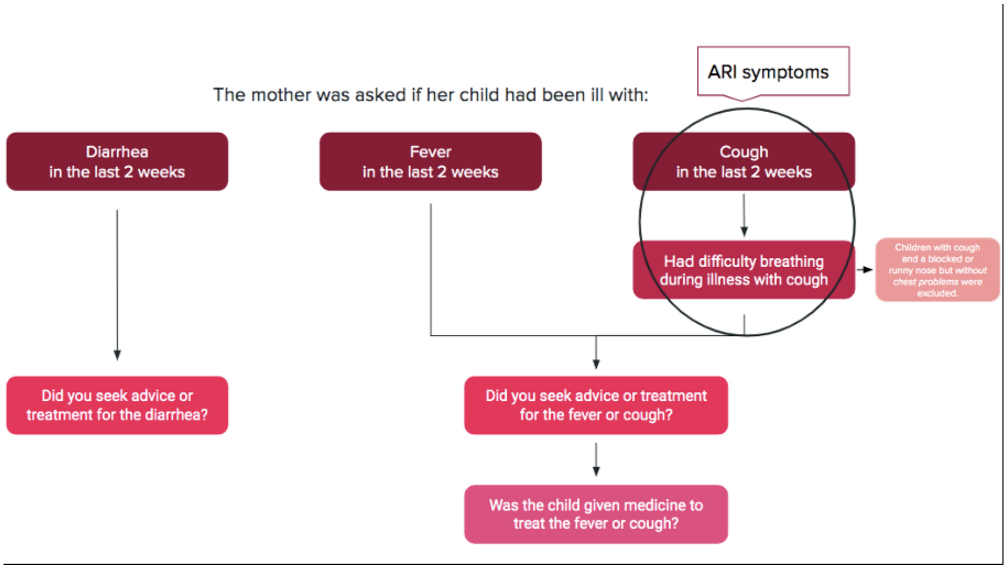

Figure 2 - Identifying episodes of illness, healthcare seeking and access to treatment

In the survey's Care of Illness (CA) module, the mother/caretaker was asked if the child had been ill with diarrhea, fever or cough in the last 2 weeks. If the child had been ill with cough, the mother/caretaker was asked if the child had difficulty breathing during that illness. The child was considered having ARI symptoms if it had cough and difficulty breathing at the same time, meaning cough due to a chest problem. To identify healthcare seeking behavior, the mother/caretaker was asked if they had sought advice or treatment for the illnesses respectively. To identify access to treatment, the mother/caretaker was asked if the child had been given medicine for the fever or cough.

160x89mm (144 x 144 DPI)

BMJ Paediatrics Open

The occurrence of major infectious diseases and health care seeking among young children with disabilities in Sierra Leone using cross-sectional population-based survey data

Journal:	*BMJ Paediatrics Open*
Manuscript ID	bmjpo-2023-002460.R1
Article Type:	Original research
Date Submitted by the Author:	14-Apr-2024
Complete List of Authors:	Ekman, Anna-Theresia; Karolinska Institutet, Global Public Health<br>Cherry, Esagila; Linkoping University Department of Health Medicine and Caring Sciences<br>Sengeh, Paul; FOCUS1000<br>Webber, Nance; FOCUS1000<br>Jalloh, Mohammad; FOCUS1000<br>Orsini, Nicola; Karolinska Institutet, Global Public Health<br>Alfvén, Tobias; Karolinska Institutet, Global Public Health<br>Frielingsdorf Lundqvist, Helena; Linkoping University Department of Health Medicine and Caring Sciences
Keywords:	Epidemiology, Mortality, Neurology

SCHOLARONE™
Manuscripts

I, the Submitting Author has the right to grant and does grant on behalf of all authors of the Work (as defined in the below author licence), an exclusive licence and/or a non-exclusive licence for contributions from authors who are: i) UK Crown employees; ii) where BMJ has agreed a CC-BY licence shall apply, and/or iii) in accordance with the terms applicable for US Federal Government officers or employees acting as part of their official duties; on a worldwide, perpetual, irrevocable, royalty-free basis to BMJ Publishing Group Ltd ("BMJ") its licensees and where the relevant Journal is co-owned by BMJ to the co-owners of the Journal, to publish the Work in this journal and any other BMJ products and to exploit all rights, as set out in our licence.

The Submitting Author accepts and understands that any supply made under these terms is made by BMJ to the Submitting Author unless you are acting as an employee on behalf of your employer or a postgraduate student of an affiliated institution which is paying any applicable article publishing charge ("APC") for Open Access articles. Where the Submitting Author wishes to make the Work available on an Open Access basis (and intends to pay the relevant APC), the terms of reuse of such Open Access shall be governed by a Creative Commons licence – details of these licences and which Creative Commons licence will apply to this Work are set out in our licence referred to above.

Other than as permitted in any relevant BMJ Author's Self Archiving Policies, I confirm this Work has not been accepted for publication elsewhere, is not being considered for publication elsewhere and does not duplicate material already published. I confirm all authors consent to publication of this Work and authorise the granting of this licence.

Confidential: For Review Only

The occurrence of major infectious diseases and health care seeking among young children with disabilities in Sierra Leone using cross-sectional population-based survey data

Abstract

Background: Children with disabilities are at risk of worse health outcomes compared to children without functional difficulties. Sierra Leone has one of the world's highest prevalences of functional difficulties among children, but little is know about the co-occurrence of major infectious diseases and health care seeking behaviours among children with disabilities.

Methods: We used household survey cross-sectional data on children two to four years old and logistic regression models estimating odds ratios (ORs) between functional difficulties and symptoms of infectious diseases including diaorrhea, fever and acute respiratory infection, adjusted for sex, age, and stunting. We also examined whether caregivers sought advice or treatment for the illness from any source and if the child was given any treatment for the illness.

Results: There was an increased risk of fever among children with functional difficulty (AOR = 1.3, 95% CI 1.1 - 1.8) and children with severe functional difficulty (AOR = 1.6, 95% CI 1.0-2.7). Children with severe functional difficulty were also at increased risk of diarrhea (AOR = 1.8, 95% CI = 1.1 – 3.3). There were no significant differences in seeking advice or treatment for diarrhea, fever or ARI symptoms between the groups.

Conclusions: In Sierra Leone, children with functional difficulties, especially severe functional difficulties, more often have symptoms of major childhood diseases that are known to increase under-five mortality.

Key message

What is already known on this topic: Functional difficulty among young children is common in Sierra Leone. Even so, there is lacking information on major infectious diseases and health care seeking among children with functional difficulties in Sierra Leone.

What this study adds: We found that the odds of fever was 30% higher among children with functional difficulty (AOR = 1.3, 95% CI 1.1 - 1.8), and 60% higher among children with

Confidential: For Review Only

severe functional difficulty (AOR = 1.6, 95% CI 1.0-2.7). For children with severe functional difficulties, it was also more common with diarrhea (AOR = 1.8, 95% CI = 1.1 – 3.3).

How this study might affect research, practice or policy: To overcome these health inequities, efforts must be made to include children with disabilities in preventive and health promoting child health as well as curative care, while acknowledging that targeted interventions tailored to the needs of children with disabilities will be of additional benefit.

Confidential: For Review Only

Introduction

Almost 240 million children worldwide have functional difficulties, with the highest prevalence in West Africa [1]. Persons with disability experience significantly poorer health than their non-disabled peers, ultimately risking a shortened lifespan [2-5]. To meet the 2030 Agenda pledge of leaving no one behind [6], an increased understanding of health and health care seeking behavior among children with disabilities is needed.

Five million children in the world still die before their fifth birthday, with pneumonia, diarrhea, and malaria being the leading causes of under-five mortality after the neontal period [7]. All conditions are largely preventable and treatable through cost-effective measures, and malnutrition is an important risk factor [7].

In Sierra Leone, the under-five mortality rate is 109 deaths per 1000 live births [8]. Malnutrition is widespread, with 30% of children below five being stunted, 5% being wasted, and 14% being underweight in 2019 [9]. Mothers and caretakers of young children who are suspected of having a disease normally seek health care from sources such as community health providers. The lack of health information on children living with disabilities in Sierra Leone has previously been noted [10].

In 2010 the Government introduced 'The Sierra Leone Free Healthcare Initiative' to ensure universal health coverage by providing free preventive and curative health services for pregnant women, lactating mothers and children under 5 years of age [11]. An updated version of the policy, issued post Ebola, makes some provision for services to disabled persons, such services will also cover drugs and consultations. However, the full extent extent of such services is not specified.

The child functioning module (CFM) was introduced during the 2017 Sierra Leone Multiple Indicator Cluster Survey (MICS), as a novel tool to collect data on functional difficulties among children. In line with the biopsychosocial model, the CFM collects data on functional difficulties rather than specific biomedical impairments. The underlying impairments captured by the tool are heterogeneous and include both mild to severe impairments. The 2017 Sierra Leone MICS also provides information on leading causes of under-five mortality, measured

Confidential: For Review Only

through episodes of fever (proxy for malaria), acute respiratory infection (ARI) symptoms (proxy for pneumonia) and diarrhea [12].

Enhancing knowledge on the relationship between disability and leading causes of under five mortality can inform health planning policy to accurately reflect the needs of children, ultimately improving life length and quality for a vulnerable population. The aim of this article is therefore to examine the association between three symptoms of infectious diseases and disability among young children in Sierra Leone, compared to children without disabilities. A secondary aim is to study health care seeking behavior and access to treatment for the same symptoms in the same population.

Methods

Study design and description of the data set

This study utilizes two staged cluster-survey data from the 2017 Sierra Leone MICS [13]. The survey included 11,774 children under five of whom 11,764 mothers/caretakers were interviewed (response rate 99.9%) [14]. Data on functional difficulties was collected from children two to four years and therefore our study population was limited to children within this age span, in total 7117 children. Sample allocation and participant selection have been described in detail [14]. Data were extracted from the dataset for children under five, including information about functional difficulties from the CFM (Figure 1), and information about disease episodes, care-seeking, and access to treatment from the Care of Illness module (Figure 2).

Operationalizing disability

The CFM estimates children at risk of disability through a Likert-like scale, collecting information on functional difficulties in the functional areas of seeing, hearing, mobility, dexterity, communication, learning, behavior, and playing, using primary caregiver (usually mothers) as proxy-responders [15]. Children with functional difficulties were defined as those with a lot of difficulties and/or who cannot function at all in one or more of the different domains of functioning (Figure 1). A child with severe functional difficulty was defined as ‘cannot do at all’ in any of the domains. The only exception was the domain of controlling behaviour, where a child was considered having a functional difficulty at the response of ‘A lot more’.

Confidential: For Review Only

Episodes of diarrhea, fever or respiratory difficulties

The caregiver was asked to estimate whether the child had an episode of diarrhea, fever, or ARI symptoms during the preceding two-week period. These survey questions have previously been utilized in analyses of MICS surveys [16 17]. ARI symptoms were defined as rapid or difficult breathing during illness with cough, when symptoms were perceived to be due to a problem in the chest or both a problem in the chest and a blocked or runny nose [14 18]. Thus, only having symptoms of an upper respiratory tract infection were not considered as ARI symptoms. If the child had either diarrhea, fever or ARI symptoms, the caregiver was asked if they had sought any advice or treatment for the illness from any source and if the child was given any medicine to treat the illness (Figure 2).

Statistical Analysis

Functional difficulty/severe functional difficulty were used as independent variables. Potential predictors considered were child age, child gender, stunting, household wealth, maternal level of education, and rural-urban residence status. Crude (OR) and adjusted odds ratios (AOR) with 95% confidence intervals (CI) were estimated with multivariable logistic regression models. Stata version 16.0 was used for all survey analyses with the stratum variable from the household dataset and centering single units.

Ethical Approval and Consent to Participate

Ethical approval for data collection was attained from the Ethics and Scientific Review Committee in Sierra Leone by the Sierra Leone MICS team [14]. Informed consent was obtained from all participants during data collection, and data has been de-identified for confidentiality. This study is a secondary analysis of anonymized data from MICS, publicy accessible for research purposes through the MICS website.

Public involvement

Due to this being a secondary data analysis, it was not possible to involve the public in the design, conduct, reporting, or dissemination plans of our research.

Results

The weighted sample included 7,090 children age 2-4 years, of which 7% were identified as having functional difficulties and almost 2% were identified as having severe functional difficulties (Table 1). One in five children (20%) had fever in the last 2 weeks, 7% had

Confidential: For Review Only

diarrhea and 2% had ARI symptoms. Functional difficulty was associated with child age, child gender and stunting, and these factors were included in the adjusted logistic regression. The odds of fever was 30% higher among children with functional difficulty (AOR = 1.3, 95% CI 1.1 - 1.8) (Table 2). The odds of fever was 60% higher among children with severe functional difficulty (AOR = 1.6, 95% CI 1.0-2.7). For children with severe functional difficulties, it was also significantly more common with diarrhea (AOR = 1.8, 95% CI = 1.1 – 3.3). There was no significant differance for ARI symptoms after adjusting for confounding.

For more than two thirds of the children, caregivers had sought advice or treatment, and almost all were given some form of medicine for fever or cough. There were no significant differences in seeking advice or treatment for diarrhea, fever or ARI symptoms (Table 3). However, there was a trend towards less health care seeking for diarrhea in children with functional difficulties and severe difficulties, and a trend towards more health care seeking for fever or ARI sympoms (Table 3). In this material, there were no differences between children with or without functional difficulties in odds of being given medicine to treat the illness.

Confidential: For Review Only

Table 1 – Weighted sample characteristics of the study population 2- to 4-year-olds.

**Table 1. Demography of study population (weighted)**	
Variable	Total n (%)
Total number of children	7090 (100%)
Children with functional difficulties	471 (7%)
Children with severe functional difficulties	126 (2%)
**Child factors**	
**Sex**	
Male	3504 (59)
Female	3586 (51)
**Age**	
2 years	2388 (34)
3 years	2351 (33)
4 years	2351(33)
**Malnutrition**	
Underweight	760 (11)
Stunted	2121 (30)
Wasted	221 (3)
**Caregiver factors**	
**Education level**	
Pre-primary or none	4528 (64)
Primary	853 (12)
Junior Secondary	875 (12)
Senior Secondary or Higher	834 (12)
**Household and environmental factors**	
**Area**	
Urban	2663 (38)
Rural	4426 (62)
**Wealth**	
Poorest	1679 (24)
Second	1595 (23)
Middle	1482 (21)
Fourth	1222 (17)
Richest	1112 (16)
**Common childhood illnesses**	
Occurrence of illness during the preceding two weeks	
Diarrhoea	480 (7)
Fever	1460 (21)
**Cough**	1157 (16)
**Difficulty breathing during illness with cough**	452 (6)

Confidential: For Review Only

**Acute respiratory infection**	**111 (2)**
Health care seeking behaviour	
Sought advice or treatment for diarrhoea from any source	336 (70)
**Sought advice or treatment for the fever or acute respiratory infection**	**1070 (72)**
Access to treatment	
Given any type of treatment for diarrhoea	414 (86)
**Given medicine to treat the fever or acute respiratory infection**	**1348 (91)**

Confidential: For Review Only

Table 2 - Weighted odds ratios of infectious diseases in children with and without functional difficulty.

Table 2. Weighted odds ratios of infectious diseases in children with and without functional difficulty				
	Functional difficulties		Severe functional difficulties	
	Odds ratio (95% CI*)	Adjusted** odds ratio (95% CI)	Odds ratio (95% CI)	Adjusted** odds ratio (95% CI)
Occurrence of illness during the preceding two weeks				
Diarrhoea	1.4 (1-2)	1.3 (.9 - 1.8)	2 (1.1- 3.6)	1.8 (1 - 3.3)
Fever	1.4 (1-1.9)	1.3 (1 - 1.8)	1.7 (1 - 2.7)	1.6 (1 - 2.7)
Cough	1 (.8-1.4)	.9 (.7 - 1.3)	2 (1.2 - 3.5)	2 (1.1 - 3.3)
Difficulty breathing during illness with cough	1.4 (1-2)	1.3 (.9 - 2)	1.7 (.8 - 3.8)	1.7 (.7 - 3.7)
Acute respiratory infection	1.8 (1 - 3.5)	1.7 (.9 - 3.3)	2.6 (.9 - 7.2)	2.4 (.8 - 7)

*CI = Confidence Intervals. ** Adjusted for child's age, sex, and stunting.

Table 3 – Weighted odds ratios of health care seeking behaviour in children with and without functional difficulty.

Table 3. Weighted odds ratios of health care seeking behaviour in children with and without functional difficulty				
	Functional difficulties		Severe functional difficulties	
	Odds ratio (95% CI*)	Adjusted** odds ratio (95% CI)	Odds ratio (95% CI)	Adjusted** odds ratio (95% CI)
Health care seeking behaviour				
Sought advice or treatment for diarrhoea from any source	.8 (.4 -1.6)	.8 (.4 - 1.6)	.9 (.3-3)	.9 (.3 - 3.1)
Sought advice or treatment for the fever or acute respiratory infection	1.2 (.7 - 2.1)	1.2 (.7 - 2)	1.3 (.5 - 3.7)	1.3 (.5 - 3.8)
Access to treatment				
Given any type of treatment for diarrhoea	.8(.3-1.9)	1 (.4 - 2.5)	1 (.2 - 5)	1.3 (.2 - 6.6)
Given medicine to treat the fever or acute respiratory infection	1 (.5 - 2.2)	1.1 (.6 - 2.4)	1.1 (.3 - 4.3)	1.3 (.3 - 4.7)

*CI = Confidence Intervals. ** Adjusted for child's age, sex, and stunting.

Confidential: For Review Only

Discussion

In this study, children with functional difficulties were more prone to experience episodes of fever, and children with severe functional difficulties were significantly more likely to be ill with diarrhea, compared to children without functional difficulty. We found no significant difference on seeking or accessing treatment between children with or without functional difficulties.

Several conditions leading to functional difficulties, such as autism spectrum disorder, ADHD, cerebral palsy, and seizures/epilepsy, are associated with somatic, psychiatric, and neurological comorbidities [19]. For example, children with cerebral palsy often have dysphagia, which places them at risk for aspiration pneumonia [20]. This may be part of the reason behind the association between functional difficulty and the examined symptoms and infectious diseases.

Even though it is not possible to understand the underlying impairments it is likely that children with severe functional difficulties have more outspoken conditions, such as severe cerebral palsy. Indeed, a recent article investigating the same study population found a high risk of co-morbidity between difficulties in different functional domains [21], which reflects that some children within the group are more affected than others.

Another possible mechanism of association may be malnutrition. The relationship between malnutrition and childhood disability has been confirmed in multiple studies [22], and malnutrition is a shared risk factor for pneumonia, diarrhea and malaria [23]. It has also been established that children with functional difficulties in Sierra Leone have an increased level of stunting of 38% [21]. While this could explain part of the seen relationship between disability and infectious diseases, the increased odds of symptoms of infectious diseases persist after adjusting for stunting in this study population. The plethora of biomedical conditions captured by the CFM makes it difficult to fully entangle the relationship between disability, infectious diseases and malnutrition.

The increased susceptibility to major infectious diseases among children with disability has previously been described. A multilateral analysis of three to four-year-old's at risk for intellectual disability utilizing MICS data from 2010 to 2015 and data from 24 low- and middle-income countries, found an increased risk of symptoms of ARI, diarrhea, and fever [17].

Confidential: For Review Only

Although it did not comment on country-specific results, estimates for Sierra Leone were reported, showing that children at-risk of intellectual disability were significantly less likely to have diarrhea [17]. Our results align with the study's pooled findings for low-income countries, but differ from the study's findings on Sierra Leone.

A secondary finding is that one in five children in Sierra Leone had fever during the previous two weeks, likely reflecting that fever is an unprecise proxy for malaria. The high proportion in this survey is in line with results from similar surveys [16].

Strengths and Limitations

This study builds on a large and nationally representative study population, and the introduction of the CFM may allow for comparison between settings and to repeat measures in similar study populations over time. However, cross-sectional data cannot be used to draw conclusions about causality, and it is not possible to know what types of impairments underpin the individual child's functional difficulties.

The proxies used for the infectious illnesses may have low specificity; it has been described that not all reported ARI symptoms are in fact true pneumonia [18] and fever can be caused by other diseases than malaria. In some surveys a rapid diagnostic test for malaria is included, but this was not the case in the Sierra Leone 2017 MICS. However, all disease proxies used are well-established in household surveys. Also, the fact that caregivers report symptoms from the two weeks preceding the survey, makes it likely that estimated prevalences are susceptible to seasonal changes in disease patterns. This study focuses on the difference between groups of children which we believe are less affected by seasonal variation.

We could not document significant differences on health care seeking behavior or access to treatment. While this may be true, it can also be due to the relatively small number of children with functional difficulties in the dataset who had symptoms of each infectious disease. This was also the reason why the multilateral analysis mentioned above could not generate country-specific estimates of access to treatments [17].

Conclusion

In Sierra Leone, children with functional difficulties, especially severe functional difficulties, are more affected by major childhood diseases that are known to increase under five

Confidential: For Review Only

mortality. To overcome these health inequities, effort must be made to include children with disabilities in preventive and health promoting child health as well as curative care, while acknowledging that targeted interventions tailored to the needs of children with disabilities will be of additional benefit. Investing in health equity could result in lowered child morbidity and mortality, ultimately fulfilling the 2030 Agenda's pledge of leaving no one behind.

Funding statement: This work was supported by Swedish regional ALF funding 2019. ALF is the abbreviation in Swedish of an agreement between the central government and seven regions on physician education and clinical research. The data collection was done through the Multiple Indicator Cluster Survey and not funded through the study.

Competing interest statement: HF has received ALF funding for other studies 2022. Otherwise, none to declare.

Authors contribution: AE and EC contributed equally to the work. AE, EC, and HF conceptualized the idea and designed the study. PS, NW, MBH, NO, and TA provided input on the proposed research questions as well as methodology. All authors contributed to the subsequent drafts. All authors approved the final manuscript as submitted and agreed to be accountable for all aspects of the work.

Data sharing statement: Data are available through the UNICEF multiple indicator cluster survey webpage (https://mics.unicef.org/).

References

1. United Nations Children's Fund (UNICEF). Seen, Counted, Included: Using data to shed light on the well-being of children with disabilities. New York: UNICEF, 2021.
2. Namaganda LH, Almeida R, Kajungu D, et al. Excessive premature mortality among children with cerebral palsy in rural Uganda: A longitudinal, population-based study. *PLoS One* 2020;15(12):e0243948. doi: 10.1371/journal.pone.0243948 [published Online First: 20201229]
3. Abuga JA, Kariuki SM, Kinyanjui SM, et al. Premature mortality in children aged 6-9 years with neurological impairments in rural Kenya: a cohort study. *Lancet Glob Health* 2019;7(12):e1728-e35. doi: 10.1016/s2214-109x(19)30425-5 [published Online First: 20191022]
4. Emerson E, Savage A, Llewellyn G. Prevalence of underweight, wasting and stunting among young children with a significant cognitive delay in 47 low-income and middle-income countries. *J Intellect Disabil Res* 2020;64(2):93-102. doi: 10.1111/jir.12698 [published Online First: 2019/12/18]
5. Emerson E, Hatton C, Baines S, et al. The physical health of British adults with intellectual disability: cross sectional study. *Int J Equity Health* 2016;15:11. doi: 10.1186/s12939-016-0296-x [published Online First: 20160120]

Confidential: For Review Only

6. UN General Assembly. Transforming our world: the 2030 Agenda for Sustainable Development [Internet]. New York: United Nations, 2015.
7. United Nations Inter-agency Group for Child Mortality Estimation (UN IGME). Levels & Trends in Child Mortality: Report 2022, Estimates developed by the United Nations Inter-agency Group for Child Mortality Estimation. New York: United Nations Children's Fund, 2023.
8. World Bank. Mortality rate, under-5 (per 1,000 live births) [Internet]: The World Bank Group; [updated 2019; cited 2021 Dec 12]. Available from: https://data.worldbank.org/indicator/SH.DYN.MORT.
9. Statistics Sierra Leone (Stats SL) and ICF. Sierra Leone Demographic and Health Survey 2019 [Internet]. Freetown, Sierra Leone, and Rockville, Maryland, USA: Stats SL and ICF, 2020.
10. Kabia F, Tarawally U. Sierra Leone 2015 Population and Housing Census - Thematic Report on Disability. Freetown: Statistics Sierra Leone, 2017.
11. Witter S, Brikci N, Harris T, et al. The Sierra Leone Free Health Care Initiative (FHCI) process and effectiveness review. HEART (Health & Education Advice & Resource Team), 2016.
12. World Health Organization. WHO Guidelines for malaria [Internet]. Geneva: World Health Organization, 2021.
[dataset]13. Statistics Sierra Leone. Data from: Sierra Leone Multiple Indicator Cluster Survey 2017. Global MICS Programme, September 15 2021. https://mics.unicef.org/surveys
14. Statistics Sierra Leone. Sierra Leone Multiple Indicator Cluster Survey 2017, Survey Findings Report. Freetown, Sierra Leone: Statistics Sierra Leone, 2018.
15. Loeb M, Mont D, Cappa C, et al. The development and testing of a module on child functioning for identifying children with disabilities on surveys. I: Background. *Disabil Health J* 2018;11(4):495-501. doi: 10.1016/j.dhjo.2018.06.005 [published Online First: 2018/07/18]
16. Simen-Kapeu A, Bogler L, Weber A-C, et al. Prevalence of diarrhoea, acute respiratory infections, and malaria over time (1995-2017): A regional analysis of 23 countries in West and Central Africa.pdf>. *Journal of Global Health* 2021;11 doi: 10.7189/jogh.11.13008
17. Emerson E, Savage A. Acute respiratory infection, diarrhoea and fever in young children at-risk of intellectual disability in 24 low- and middle-income countries. *Public Health* 2017;142:85-93. doi: 10.1016/j.puhe.2016.10.014 [published Online First: 2017/01/07]
18. Campbell H, El Arifeen S, Hazir T, et al. Measuring coverage in MNCH: challenges in monitoring the proportion of young children with pneumonia who receive antibiotic treatment. *PLoS Med* 2013;10(5):e1001421. doi: 10.1371/journal.pmed.1001421 [published Online First: 20130507]
19. Akmatov MK, Ermakova T, Bätzing J. Psychiatric and Nonpsychiatric Comorbidities Among Children With ADHD: An Exploratory Analysis of Nationwide Claims Data in Germany. *J Atten Disord* 2021;25(6):874-84. doi: 10.1177/1087054719865779 [published Online First: 20190731]
20. Arvedson JC. Feeding children with cerebral palsy and swallowing difficulties. *Eur J Clin Nutr* 2013;67 Suppl 2:S9-12. doi: 10.1038/ejcn.2013.224
21. Ekman AT, Sengeh PA, Webber N, et al. Prevalence of children under five with disabilities in Sierra Leone in 2017: Insights from a population-based multiple indicator cluster survey. *Disabil Health J* 2023:101481. doi: 10.1016/j.dhjo.2023.101481 [published Online First: 20230504]

Confidential: For Review Only

22. Hume-Nixon M, Kuper H. The association between malnutrition and childhood disability in low- and middle- income countries: systematic review and meta-analysis of observational studies. *Trop Med Int Health* 2018;23(11):1158-75. doi: 10.1111/tmi.13139 [published Online First: 2018/08/29]
23. United Nations Inter-agency Group for Child Mortality Estimation (UN IGME). Levels & Trends in Child Mortality: Report 2020 [Internet]. New York: United Nations Children's Fund, 2020.

Figure Legend

Figure 1 - Identifying children with functional difficulties
The child functioning module asked the mother/caretaker a set of questions on eight areas of functioning regarding their child. Each area was assessed against a rating scale, with the answer categories 'No difficulty', 'Some difficulty', 'A lot of difficulty' and 'Cannot do at all'. The child was considered having a functional difficulty at the response of 'A lot of difficulty' or 'Cannot do at all', and severe functional difficulty at the response of 'Cannot do at all'. The only exception was the domain of controlling behavior, where the response categories were instead 'Not at all', 'Less', 'The same', 'More' and 'A lot more' (not presented in the figure). The child was considered having a functional difficulty at the response of 'A lot more'.

Figure 1 - Identifying episodes of illness, healthcare seeking and access to treatment
In the survey's Care of Illness (CA) module, the mother/caretaker was asked if the child had been ill with diarrhea, fever or cough in the last 2 weeks. If the child had been ill with cough, the mother/caretaker was asked if the child had difficulty breathing during that illness. The child was considered having ARI symptoms if it had cough and difficulty breathing at the same time, meaning cough due to a chest problem. To identify healthcare seeking behavior, the mother/caretaker was asked if they had sought advice or treatment for the illnesses respectively. To identify access to treatment, the mother/caretaker was asked if the child had been given medicine for the fever or cough.

Confidential: For Review Only

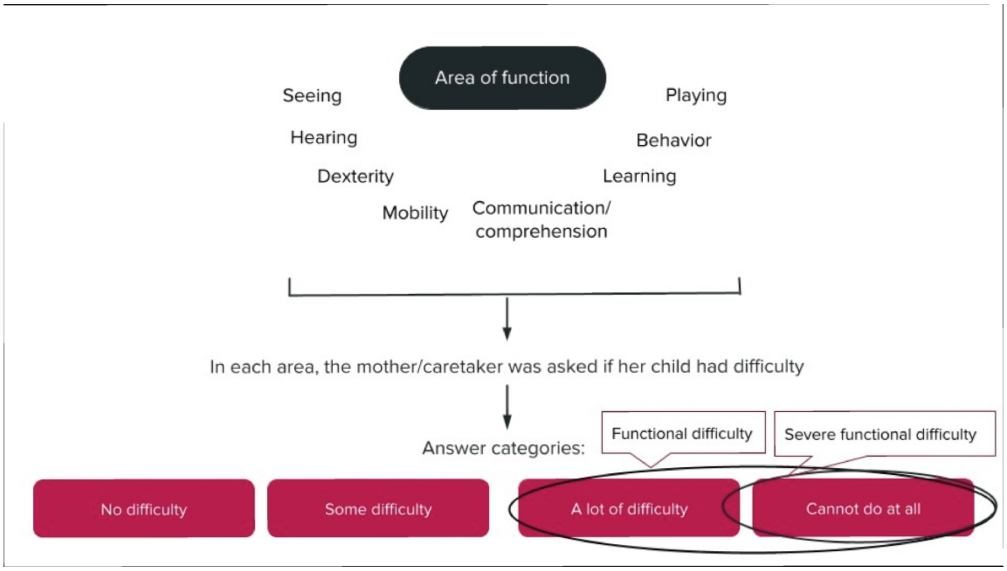

Figure 1 - Identifying children with functional difficulties

The child functioning module asked the mother/caretaker a set of questions on eight areas of functioning regarding their child. Each area was assessed against a rating scale, with the answer categories 'No difficulty', 'Some difficulty', 'A lot of difficulty' and 'Cannot do at all'. The child was considered having a functional difficulty at the response of 'A lot of difficulty' or 'Cannot do at all', and severe functional difficulty at the response of 'Cannot do at all'. The only exception was the domain of controlling behavior, where the response categories were instead 'Not at all', 'Less', 'The same', 'More' and 'A lot more' (not presented in the figure). The child was considered having a functional difficulty at the response of 'A lot more'.

338x191mm (300 x 300 DPI)

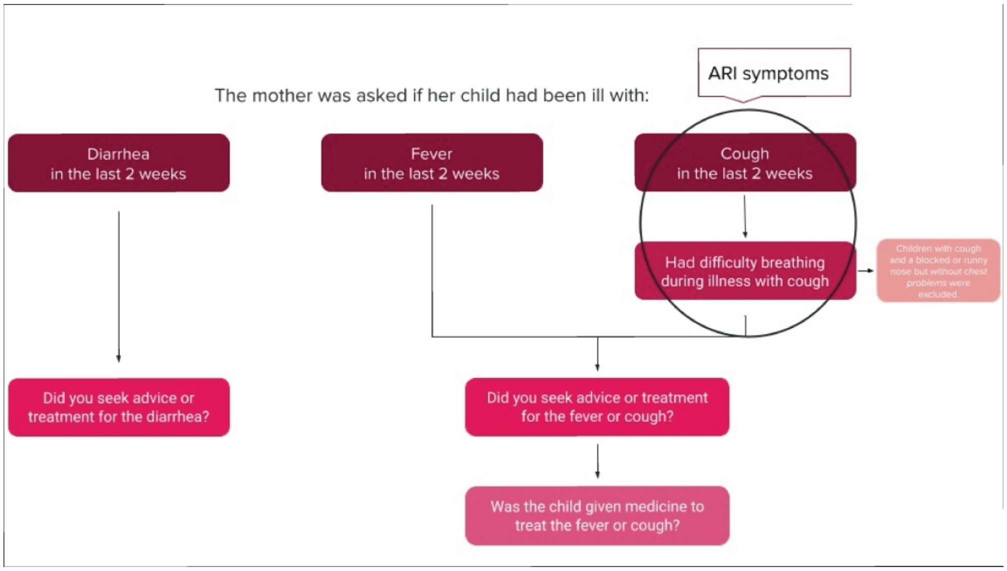

Figure 2 - Identifying episodes of illness, healthcare seeking and access to treatment

In the survey's Care of Illness (CA) module, the mother/caretaker was asked if the child had been ill with diarrhea, fever or cough in the last 2 weeks. If the child had been ill with cough, the mother/caretaker was asked if the child had difficulty breathing during that illness. The child was considered having ARI symptoms if it had cough and difficulty breathing at the same time, meaning cough due to a chest problem. To identify healthcare seeking behavior, the mother/caretaker was asked if they had sought advice or treatment for the illnesses respectively. To identify access to treatment, the mother/caretaker was asked if the child had been given medicine for the fever or cough.

338x190mm (300 x 300 DPI)
